# Utilization of Spent Coffee Grounds for Bioelectricity Generation in Sediment Microbial Fuel Cells

**DOI:** 10.3390/microorganisms12030618

**Published:** 2024-03-19

**Authors:** Nurfarhana Nabila Mohd Noor, Ilwon Jeong, Seokjin Yoon, Kyunghoi Kim

**Affiliations:** 1 Department of Ocean Engineering, Pukyong National University, Busan 48513, Republic of Korea; farhanabilla@pukyong.ac.kr; 2 Graduate School of Advanced Science and Engineering, Hiroshima University, Higashihiroshima 739-8527, Japan; 3 Dokdo Fisheries Research Center, National Institute of Fisheries Science, Pohang 37709, Republic of Korea

**Keywords:** sediment microbial fuel cells, coffee waste, caffeine concentration, bioelectricity generation, coastal sediment

## Abstract

This study examined the utilization of spent coffee grounds with different aqueous extraction methods for the bioelectricity generation from coastal benthic sediment through a sediment microbial fuel cell (SMFC) system. Different methods for the aqueous extraction of SCGs were evaluated, including rinsing and drying of the SCG (SMFC-CRD), immersion, rinsing and drying (SMFC-CRID), drying alone (SMFC-CD), and untreated SCG (SMFC-C). The caffeine concentration in the SCG was significantly reduced using pretreatments, with SMFC-CRID achieving the lowest concentration of 0.021 ± 0.001 mg/g. SMFC-CRD contributed to the generation of the highest current density of 213.7 mA/m^2^ during closed-circuit operation and exhibited the highest power density of 96.9 mW/m^2^ in the polarization test, due to the suitable caffeine content of 0.275 ± 0.001 mg/g in the SCG. This study could provide a cost-effective method for reusing SCGs (i.e., 128 g) while generating bioelectricity as an alternative energy source. These results suggest that pretreatment with SCGs is essential for achieving optimal power density and reducing the caffeine concentration in the SMFC system.

## 1. Introduction

Coffee is an important product that contributes to the economic growth and financial improvement of countries. In recent years, South Koreans have significantly increased their daily coffee consumption to 12 times a week [1]. However, the high coffee consumption results in a large amount of untreated coffee waste, also known as spent coffee grounds (SCGs), due to the lack of practical disposal methods. The disposal of excess SCGs as solid waste in landfills contributes to soil pollution and groundwater contamination [2,3]. Compounds contained in SCGs, such as phenol, tannin, and caffeine, can leach into the environment [4] and into water bodies through soil percolation, which increases the acidity of the soil [5]. As a result of anaerobic decomposition, large amounts of methane are released into the atmosphere during this process, which accelerates global warming [6,7]. Caffeine (1,3,7-trimethylxanthine) in coffee grounds is a stable compound that can withstand high temperatures [8]. The compound remains intact because of its low ability to separate into water when being heated [9]. Therefore, the caffeine content in coffee beans remains constant regardless of the degree of roasting [8]. However, long brewing times reduce the caffeine content of SCGs as they comprise water-soluble alkaloids [10]. SCGs are usually discarded as a valueless waste since there is no specific method for removing caffeine from this waste [11]. Due to the low efficiency of caffeine-containing wastewater treatment methods, caffeine has been detected in the surface as well as groundwater [12]. Therefore, reusing SCGs using appropriate technology is essential for reducing the need for excessive waste disposal.

The extraction of electricity from organic waste through the system of sediment microbial fuel cells (SMFCs) is strongly dependent on the ability of exoelectrogens in the sediment to carry out electrochemical reactions. The SMFC system accelerates the decomposition of organic material in the sediment and generates protons and electrons through microbial metabolism activity in order to produce sustainable bioelectricity [13]. The system transfers electrons from the anode to the cathode, while the protons diffuse from the anodic region into the overlying water and eventually reach the cathodic region. In the cathodic region, the oxygen reacts with the transferred electrons and protons via an oxygen reduction reaction (ORR) to produce water as the final product of the SMFC system. The concentration of organic matter in the sediment primarily affects the overall performance of the SMFC system [14]. Improving the anodic region of the SMFC system benefits the bioremediation of contaminated sediments [13,14] and environmental monitoring [15], as the SMFC system can efficiently convert organic substrates into electricity. SCGs are a biowaste with a high content of organic matter and nutrients [4,16,17]. Therefore, it can be used to enhance the bioelectrogenesis of sediment microbes for the generation of sustainable bioelectricity.

Previous studies have investigated the use of SCGs by composting the organic wastes [18] or converting them into carbonized materials [19]. Yeo and Yang [20] used SCGs as the primary feedstock for the worm compost as an MFC anolyte. The direct application of SCGs in the sediments can affect the performance of the SMFC system by hindering power generation of the system. Beltrán et al. [21] and Nayak et al. [22] pointed out that the sensitivity to caffeine varies depending on the bacterial species and that caffeine concentrations higher than 2.5 g/L inhibit several bacterial species [23]. SCGs contain polysaccharides and phenolic compounds that can be utilized as the substrates by the sediment microbes. Consequently, SCGs could be a green option for bioelectricity generation in the SMFC systems. Previous studies on coffee in the literature on MFCs have used coffee wastewater [24,25], carbonized SCGs [19,26], and composted SCGs [27] as their substrates within their MFC systems. Considering all this information, this study aimed to evaluate the potential of pretreatment of raw SCGs via aqueous extraction methods, including the rinsing and immersion action, for bioelectricity generation in the SMFC system as well as the waste-reusing purposes since the direct use of raw SCGs in benthic coastal sediment-based fuel sources has not yet been extensively explored. The performance of the anodic biofilms was monitored and discussed in terms of their bioelectricity performances, given that the generation of bioelectricity from the SMFC system reflects the electrochemical activity of the coastal benthic sediment’s microbes.

## 2. Materials and Methods

### 2.1. Sample Preparation of Caffeine Determination

**Figure 1:**
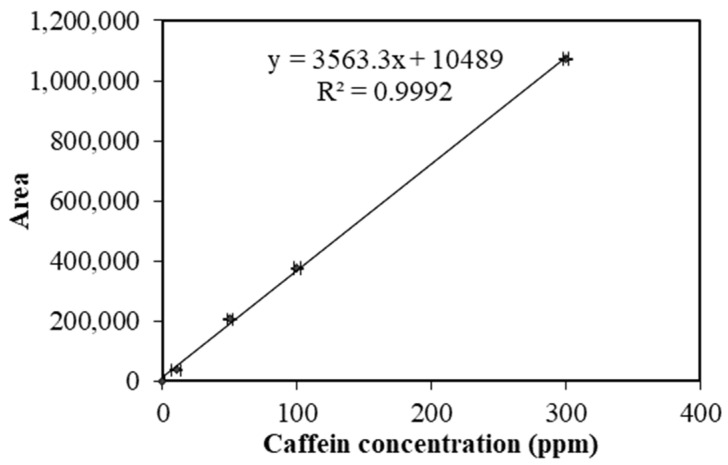
The calibration curve of the standard caffeine concentration using high performance liquid chromatography (HPLC) method.

The solvents and reagents used for liquid chromatography were of analytical grade. A caffeine stock solution (1000 ppm) was prepared in methanol to obtain a series of caffeine working solutions. In total, 50 mg of high-purity caffeine (CAS No.: 58-08-2, Sigma-Aldrich, St. Louis, MO, USA) was dissolved in 50 mL of methanol in a 50 mL volumetric flask. The working solutions of 0 ± 0.001, 10 ± 0.005, 50 ± 0.002, 100 ± 0.002, and 300 ± 0.003 ppm were prepared by a serial dilution of the stock solution in 50 mL volumetric flasks before topping to the mark with HPLC-grade ultrapure water. To determine the caffeine concentration in the SCG, an external calibration curve of peak areas was plotted against the concentration of the caffeine standards, as shown in Figure 1.

The SCG was sieved under a 355 µm mesh to maximize the extraction of caffeine. For caffeine extraction from the SCG, 1 g of raw and dried SCG was placed in 250 mL beakers after weighing them with an analytical balance. HPLC-grade ultrapure water (100 mL) was added and gently swirled for 20 min, following the usual brewing time for the coffee. The caffeine from the SCG was extracted at 25 °C in order to stimulate the natural release of caffeine at a room temperature. Using a nylon micro syringe filter (0.45 μm, Hyundai Micro, Seoul, Republic of Korea), 2 mL of the caffeine extract was filtered to obtain a transparent solution and to remove impurities as well as suspended solids for HPLC analysis. The samples were transferred to a 2 mL vial. The analysis was performed with two replicates for each experimental case of the SMFC.

The chromatographic analysis was performed using a Shimadzu HPLC system equipped with a DGU-20A degassing unit, an SPD-20A Prominence UV-VIS detector, a CTO-20A column oven, a LC-20AT liquid chromatograph, and a SIL-20A autosampler. The HPLC conditions for the quantification of caffeine concentration for SCGs were as follows: the HPLC Shimadzu (model SPD-20A) with a C18 column (5 μm particle size, 4.6 mm internal diameter and 250 mm length; WAT054275, Waters Symmetry, Sevenoaks, UK) for separation with a column temperature of 40 °C and a UV detector set at 275 nm. The sample injection volume was 10 µL and the gradient elution was performed with ultrapure water (phase A) and methanol (phase B) as mobile phases (water/methanol; 60:40) at a flow rate of 0.8 mL/min with a total run time of 20 min. The caffeine concentration in the SCG was measured using an equation derived from the linear regression of the calibration curve of standard caffeine; subsequently, the results were presented in ppm, as shown in Figure 1.

### 2.2. Preparation of Spent Coffee Grounds

SCGs were acquired at a local coffee shop near Pukyong National University, Daeyeon Campus, Busan, Republic of Korea. This study included five cases of SMFCs based on the experimental configurations of SMFCs, as presented in Table 1. The experimental cases were represented by a control case with an SMFC system without SCGs as SMFC-Cont, 5% SCG without moisture removal that was denoted as SMFC-C, 5% SCG with moisture removal that was denoted as SMFC-CD, 5% SCG thoroughly rinsed with deionized water for 15 min prior to moisture removal that was denoted as SMFC-CRD, and 5% SCG rinsed and immersed in deionized water for 12 h prior to moisture removal that was denoted as SMFC-CRID. Moreover, 32 g of the SCG (i.e., 5 % of the volume of the sediment) was mixed with 640 mL (wet volume) of the sediment. The SCGs were dried in an oven at 105 °C for 12 h to remove the moisture within them. The deionized water used for aqueous extraction was kept at a room temperature of 25 °C.

### 2.3. Establishment of SMFC Reactors

**Figure 2:**
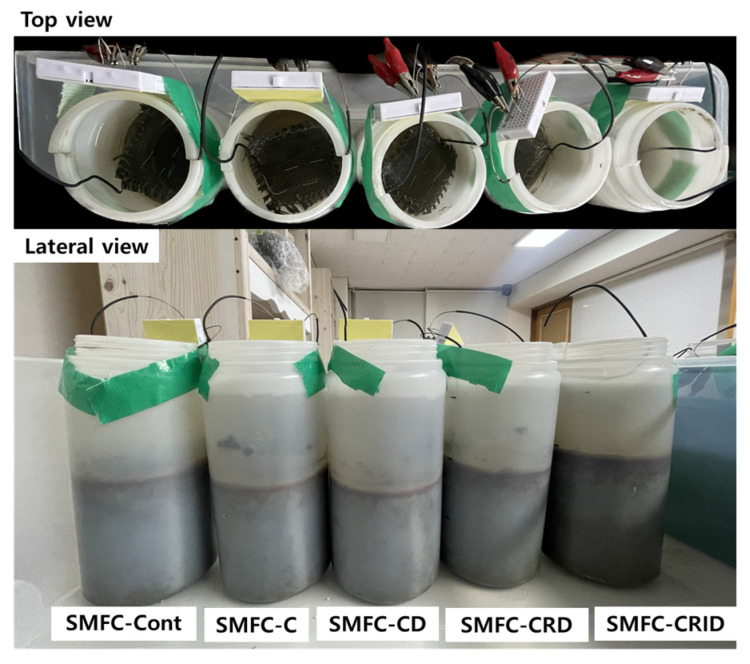
Experimental setup for the laboratory experiment of sediment microbial fuel cell (SMFC) with coffee waste (also referred to as SCG).

The experimental setup for the laboratory experiment of the SMFC with the SCG is shown in Figure 2. A carbon cloth with a diameter of 6–7 µm (E-C-CC1-06, Japanese Electro-Chem) was used as the electrode material for both the anode and cathode. The carbon cloth was cut several times to a size of 5 × 5 cm to form square-shaped electrodes. The electrodes were heated in a furnace at 500 °C for 30 min to increase their specific area and electrode performance, as described by Tran et al. [14]. Each SMFC reactor was set up in a high-density polyethylene (HDPE) cylindrical bottle (volume: 1 L; height: 20 cm; diameter: 9 cm). The SCG and sediment were thoroughly mixed in order to create a homogeneous environment in the anodic region of SMFC system [28]. The anodic region was then filled with 640 mL of sediment (height: 10 cm). The anode was embedded 5 cm below the sediment surface. The remaining sediment, 5 cm in height, was layered on top of the anode.

It was noted that the sediment microbes utilized in the SMFC system originated from the benthic coastal sediment from Sacheon, Gyeongsang Province, Republic of Korea. The anaerobic bacteria in the benthic coastal sediments convert organic waste into bioelectricity through the oxidation process [29]. During operation of the SMFC, there were no additional microbes or nutrients introduced into the sediment as an anodic region, so the system relied solely on the benthic coastal sediment as its fuel for the SMFC system. Additionally, 360 mL of deionized water was carefully added in the cathodic region in order to avoid resuspension of the sediment. The cathode was then suspended in the overlying water (height: 8 cm) after the water became clear. The deionized water was periodically added in the cathodic area in order to replace the water lost by the evaporation. A 33 cm titanium wire (φ 0.80 mm, 99.5%; TI-451465, Nilaco, Tokyo, Japan) was used as the current collector between the electrodes. The laboratory experiments of the SMFC system were operated at a room temperature of 25 °C for 14 days.

### 2.4. Electrochemical Measurements

The acclimation time for the microbial community was set to seven days. An external resistor (1000 Ω) was connected in a closed-circuit mode for 14 days to drive the electron movement and the current generation. Cell voltage was recorded every 10 min using an electrochemical workstation (WBCS3000Le; WonATech, Seoul, Republic of Korea). Current (I) was calculated using Ohm’s law, where I = V/R. Current density was calculated as I/RA, and power density was calculated as P = IV/A, where I (mA) is the current, V (V) is the voltage, R is the Ω, and A (m^2^) is the anode projected surface area.

The polarization curves were obtained by varying the applied external resistance at the end of the experiment. Both electrodes were connected to different external resistances at 10 min intervals once the voltage had stabilized. The applied external resistances were as follows: 0 Ω, 220,000 Ω, 100,000 Ω, 67,000 Ω, 51,000 Ω, 47,000 Ω, 20,000 Ω, 10,000 Ω, 6800 Ω, 5100 Ω, 4700 Ω, 3300 Ω, 2000 Ω, 1000 Ω, 680 Ω, 510 Ω, 470 Ω, 330 Ω, 200 Ω, 100 Ω, 47 Ω, and 22 Ω. To plot electrode polarization curves, the electrode potentials of the anode and cathode under a series of external resistance were measured manually using a digital multimeter (3280-10F, Hioki, Japan) against an Ag/AgCl reference electrode (CHI111, CH Instruments, Bee Cave, TX, USA). The total voltage obtained for the polarization curves was calculated using the formula of the cell potential of voltage = cathode potential − anode potential.

## 3. Results and Discussion

### 3.1. Caffeine Concentration after Simple Pretreatment

**Figure 3:**
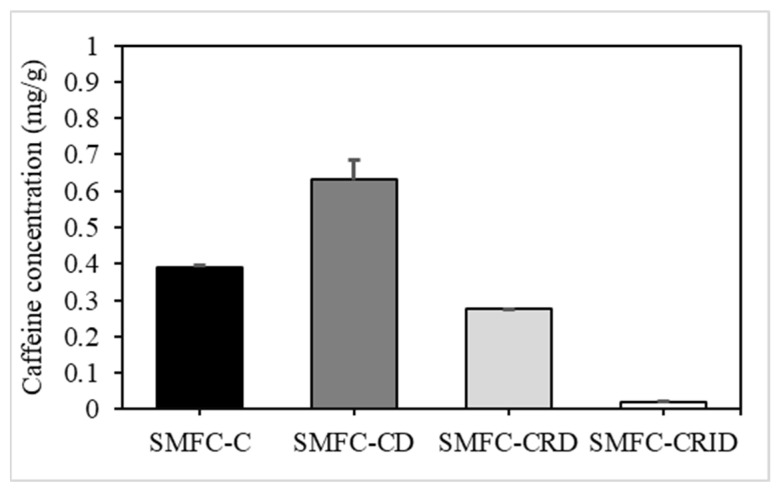
The caffeine content of the SCG in SMFC experimental cases through the high performance liquid chromatography (HPLC) method.

Figure 3 shows the caffeine content of the SCG with and without aqueous extraction in the SMFC experiments using HPLC. The lowest caffeine content was found in SMFC-CRID (0.021 ± 0.001 mg/g), followed by SMFC-CRD (0.275 ± 0.001 mg/g), SMFC-C (0.391 ± 0.055 mg/g), and SMFC-CD (0.631 ± 0.003 mg/g). This study found that the caffeine concentration of an oven-dried SCG of SMFC-CD increased from its initial concentration of 0.391 ± 0.055 mg/g to 0.631 ± 0.003 mg/g. Since caffeine resists high temperatures, this compound was able to remain intact in the SCG due to its recalcitrant nature [8]. However, after oven drying, the moisture content and total weight of the SCG decreased, which may have resulted in a higher caffeine content on a weight basis in SMFC-CD [30].

Meanwhile, the caffeine content in SMFC-CRID and SMFC-CRD was found to be significantly reduced, at 0.021 ± 0.001 mg/g and 0.275 ± 0.001 mg/g, respectively. The aqueous extraction of the SCG in SMFC-CRD and SMFC-CRID possibly reduces the toxicity of caffeine and helps to promote the production of bioelectricity in the SMFC. SMFC-CRID showed the lowest caffeine concentration among all experimental cases. The short aqueous extraction by rinsing and immersion of the SCG reduces the caffeine content as the caffeine has the ability to interact with water molecules via hydrogen bonds [31]. Water is known to be an excellent polar solvent for caffeine, as it dissolves and saturates the solvent, thereby decreasing the caffeine concentration in the SCG. Therefore, the caffeine concentration decreased more during aqueous extraction with rinsing and overnight immersion in SMFC-CRID than during rinsing alone in SMFC-CRD.

To simplify, the short aqueous extraction of the SCG in SMFC-CRD and SMFC-CRID could minimize the caffeine content for the optimal utilization of direct SCG loading in the SMFC system. It should be noted that the caffeine concentration in this study was obtained from collected coffee waste, so the caffeine content in the SCG was lower compared to normal coffee beans or ground coffee before the brewing process. Nevertheless, it should be noted that the short aqueous extraction by rinsing or immersing the SCG may not remove all the remaining caffeine from the SCG. Some caffeine may remain trapped in the SCG despite rinsing or immersion. The effectiveness of these methods to reduce caffeine content may vary and may not be complete. It is therefore suggested that the use of hot water may facilitate the extraction of caffeine from the SCG through its open pores [32].

### 3.2. Performance of Open-Circuit Voltage in SMFC Cases

**Figure 4:**
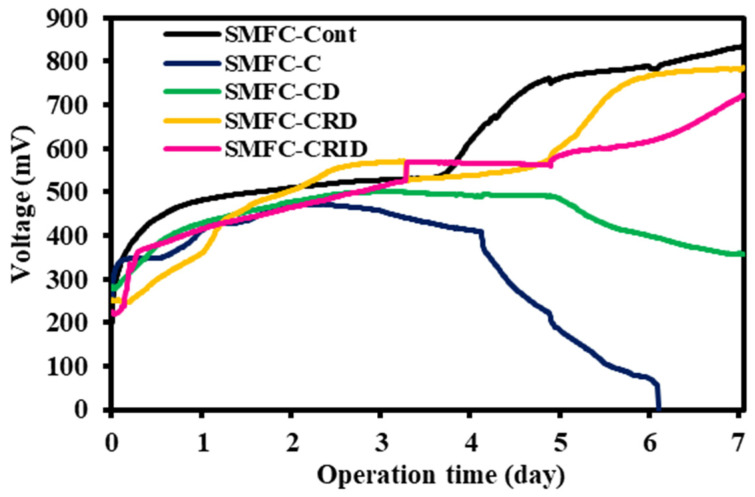
The generation of open-circuit voltage of the sediment microbial fuel cell (SMFC) systems for 7 days.

Figure 4 shows the generation of the SMFC experimental cases’ open-circuit voltage (OCV) for seven days. The OCV value represents the initial state of the fuel cell before various losses occur during the operation of the SMFC [33]. The system operates in an idle state without external resistance, which provides valuable information about the highest electrochemical potential that can be achieved by the system. The OCV may not be ideal for a direct investigation of biofilm formation or microbial activity as there is no current flowing between the anode and cathode. Nevertheless, the microbes in the sediment need to attach, grow, and adapt to the introduced anode in a natural environment to influence the electron flow. Therefore, the early adaptation could be monitored using the OCV values before the system is connected to an external resistor in closed-circuit mode.

In this study, it took about 4 days for the microbial community to acclimatize to the newly introduced anode before the OCV value increased in the experimental cases, except for SMFC-C and SMFC-CD. Meanwhile, SMFC-Cont reached the highest OCV value of 833.7 mV. On day 5, the OCV value of SMFC-CRD and SMFC-CRID increased, reaching 785.3 mV and 722.3 mV on day 7, respectively. Since the voltage is almost the same as SMFC-Cont, it might be difficult to attribute the increase in voltage on day 5 to the SCG in SMFC-CRD and SMFC-CRID. However, SMFC-CRD and SMFC-CRID showed an increase in OCV values compared to SMFC-C and SMFC-CD, which can be attributed to the decrease in the SCG’s caffeine concentration due to the removal of caffeine. The short treatment of the SCG via aqueous extraction probably triggered voltage acceleration in SMFC-CRD and SMFC-CRID. The voltage generation in SMFC-C, however, experienced a sudden decrease on day 4.

The delay in the increase of voltage in SMFC-CRD and SMFC-CRID until day 5 suggests that the biofilm required several days to acclimatize and stabilize after the addition of the SCG. From day 5, the OCV of SMFC-CD gradually decreased and reached 358.2 mV. This could be due to the increased caffeine content after oven drying the SCG in SMFC-CD (Figure 3). The OCV value of SMFC-C decreased significantly on day 4, which probably occurred due to the depletion of the compounds that serve as electron donors in SMFC. In SMFC-C, direct SCG loading in the sediment of the anodic region likely resulted in caffeine leaching into the cathodic region through sediment percolation [34]. The caffeine crossover could lead to undesired side reactions at the cathode and deteriorate the OCV value of SMFC-C [35]. Therefore, it was suggested that direct SCG loading causes an abrupt voltage drop in the SMFC-C, which could deteriorate anodic catalytic activity as well as OCV performance.

### 3.3. Bioelectricity Generation of SMFC

**Figure 5:**
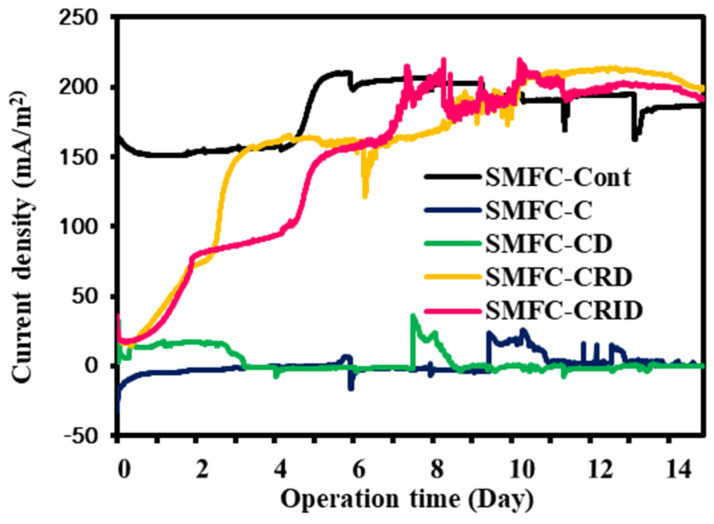
The generation of current density in the sediment microbial fuel cell (SMFC) for 14 days.

Figure 5 shows the generation of the SMFC experimental cases’ current density over 14 days. The start-up time of the SMFCs was probably influenced by the SCG pretreatment via aqueous extraction. SMFC-Cont initially remained stable between 150 mA/m^2^ and 160 mA/m^2^ but increased to 210 mA/m^2^ on day 5. However, SMFC-Cont was found to be depleted faster than SMFC-CRD and SMFC-CRID, reaching 186.9 mA/m^2^ on day 14. The current densities of SMFC-CRD and SMFC-CRID reached about 155 mA/m^2^ on the fifth day. This showed that they had longer start-up times than SMFC-Cont, which could be due to the adaptation of the biofilm to the new organic matter of the less-caffeinated SCG. This study showed that SMFC-CRD and SMFC-CRID performed better than SMFC-Cont on day 10, reaching 212.3 mA/m^2^ and 202.7 mA/m^2^ on day 12, respectively. However, the current densities of SMFC-CRD and SMFC-CRID dropped to 199.7 mA/m^2^ and 193.1 mA/m^2^ on day 14, respectively. In comparison to SMFC-Cont, a high concentration of organic matter in the SCG [4,16,17] could improve the generation of high current density in the SMFC system. This suggests that the aqueous extraction of the SCG with the integration of the SMFC system was appropriate due to the low caffeine content, as shown in SMFC-CRD and SMFC-CRID. In SMFC-C and SMFC-CD, it was found that the current densities did not recover during SMFC operation and remained below 25.87 mA/m^2^ and 36.57 mA/m^2^, respectively. The production of bioelectricity in SMFC-C was degraded by direct SCG loading of the sediment within the SMFC system.

In addition, SMFC-CD exhibited the highest caffeine concentration compared to SMFC-CRD and SMFC-CRID. Further immersion and rinsing of the SCG in SMFC-CRD and SMFC-CRID significantly reduced the caffeine content and improved the performance of the SMFC. The reduction of the caffeine content in SMFC-CRD and SMFC-CRID was attributed to the recalcitrant nature of caffeine, which has high-water solubility and a low octanol/water partition coefficient [9]. Therefore, SMFC-CRD and SMFC-CRID showed good performance in generating bioelectricity.

Based on the generated current density, the high moisture content of the SCG in SMFC-C significantly deteriorates the performance of the current and negatively affects the microbial metabolism in SMFC-C [18] as well as the electron transfer to the anode. The high moisture content of the SCG in SMFC-C can lead to the formation of leachate [34]. The aqueous extraction in SMFC-C and SMFC-CD reduced the toxicity of the SCG effluent and the potential formation of leachate by removing moisture, promoting the formation of anodic biofilm, and effectively generating bioelectricity from the SMFC system. Therefore, SMFC-CRD and SMFC-CRID maintained their performance at minimal caffeine concentrations compared to SMFC-C and SMFC-CD. The chemical compositions of the SCGs were influenced by the roasting and extraction methods. The weight loss of the SCGs was about 46%. The content of phenolic chemicals in the coffee beans decreases during the heating process [36]. SCGs contain an excellent carbon source for the sediment microbes and provides additional sugars for respiration, growth, and carbon storage [37]. The composition of soluble sugars in SCGs, such as glucose, mannose, galactose, and arabinose, can be used by sediment microbes for their metabolic processes [38]. It is suggested that the addition of SCGs with a suitable aqueous extraction as a substrate can stimulate the growth of sediment microbes within the SMFC system.

### 3.4. Polarization Behavior of SMFC

**Figure 6:**
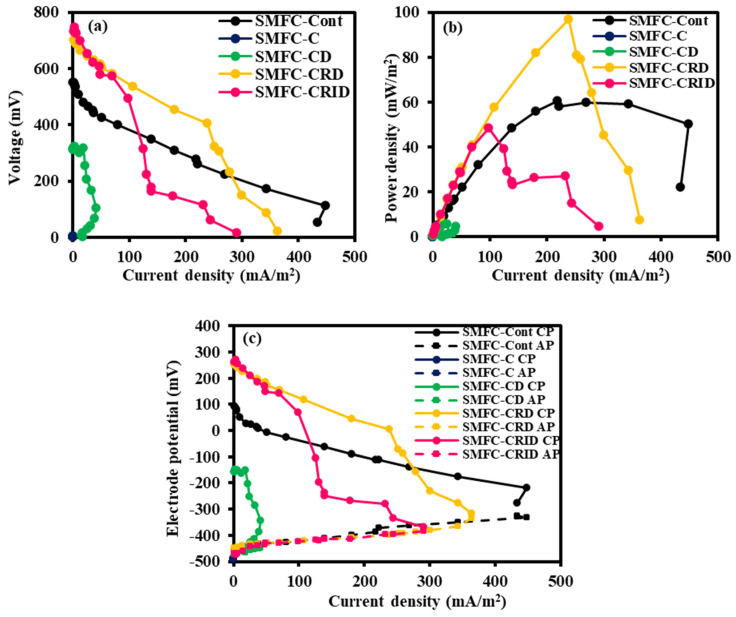
Polarization curve (**a**), power density (**b**), and (**c**) cathodic (CP) and anodic (AP) polarization (vs. Ag/AgCl electrode) as a function of current density in SMFC-Cont, SMFC-C, SMFC-CD, SMFC-CRD, and SMFC-CRID at the end of SMFC operation.

Figure 6 shows the overall performance of the SMFC after 14 days, which was evaluated using polarization curves. As can be seen in Figure 6a, SMFC-CRID exhibits the highest OCV of 732.7 mV, indicating a high electrochemical reaction rate in SMFC-CRID compared to the other experimental cases of SCG associated with the SMFC system. This was followed by SMFC-CRD (699.8 mV), SMFC-Cont (550.1 mV), SMFC-CD (311.6 mV), and SMFC-C (0 mV). SMFC-CRD achieved the highest maximum power density of 96.8 mW/m^2^ at an external resistance of 680 Ω (Figure 6b). The maximum power density occurs when the external resistance of the fuel cell is equal to its internal resistance [39]. The polarization curve showed that the pretreated SCG in SMFC-CRD performed better than SMFC-Cont, which generated 60.6 mW/m^2^ at a similar resistance of 680 Ω. SMFC-CRID and SMFC-CD achieved 48.4 mW/m^2^ (at 2000 Ω) and 6.0 mW/m^2^ (at 6800 Ω), respectively, while SMFC-C had the lowest maximum power density of 0.03 mW/m^2^. Based on Figure 6a, SMFC-Cont and SMFC-CD experienced an overshoot phenomenon, leading to a sudden drop in current density and voltage at low external resistances, which is due to electrical and ionic depletion within their SMFC systems [14,40]. This implies that their anodic region lacks electrons reaching the cathode because the electron demand at the cathode exceeds the microbial production in the anode, thus hindering the generation of high current density during the polarization test.

The results of the anodic and cathodic polarizations are shown in Figure 6c. While anode potential showed similar trends to SMFC-Cont, the differences in current density were observed in all cases. The high concentration of caffeine in SMFC-C and SMFC-CD could possibly degrade the anodic microbes as the current density generated here was the lowest compared to the other cases. Despite the direct exposures to SCGs, the sediment conditions in the less-caffeinated cases of SMFC-CRD and SMFC-CRID may not have deteriorated significantly as their anodic polarizations were comparable to those of SMFC-Cont. For SMFC-C and SMFC-CD, the generated current density deteriorated due to high caffeine content, which reduced electron transfer to the cathodic region and ORR activity. The electrode polarization tests showed that the cathodic performance also limited SMFC performance. This study showed that SMFC-C and SMFC-CD deteriorated in cathodic polarization potential, whereas SMFC-CRD and SMFC-CRID were better than SMFC-Cont. The exoelectrogens exhibit different kinetic properties when the electrons migrate from the anode to the cathode [29,41]. The stable anodic potentials of SMFC-CRD and SMFC-CRID indicated better biofilm tolerance for electron transfer kinetics to the cathodic region, resulting in a higher cathodic potential than SMFC-C and SMFC-CD.

SMFC-CRD and SMFC-CRID initially exhibited high cathodic polarizations. However, the decrease in cathodic polarization was probably due to slight caffeine interference during the ORR process. SMFC-CD exhibited a high cathodic ohmic resistance based on a steep polarization slope. If caffeine was not removed from the SCG, it could possibly diffuse into the cathodic regions of SMFC-C and SMFC-CD and, thus, delay the electron transfer for the ORR process at the cathodic region. However, the hypothesis of the diffusion of caffeine from the anodic to the cathodic region could not be clearly clarified in this study and, therefore, requires a further in-depth analysis. Yap et al. [9] showed that caffeine molecules can interfere with ORR activity in the cathodic region. Caffeine molecules bind to the hydroxyl groups in water [9,12] and thus limit the cathodic ORR in SMFC-C and SMFC-CD.

## 4. Conclusions

This study showed that the SMFC system with the use of coffee waste (also referred to as SCG) with aqueous extraction works better than without aqueous extraction. Based on the results, direct loading of the SCG is not good for the SMFC system, so aqueous extraction is required here to reduce the toxicity of caffeine from the SCG to the microbial metabolic activity in the anodic region. Therefore, the lowest caffeine content showed the highest maximum power density by reducing the caffeine concentration via a short aqueous extraction of the rinse and immersion method. The highest maximum power density of 60.6 mW/m^2^ was achieved for SMFC-CRD, which corresponds to the closed-circuit current density, and this could be attributed to a low concentration of caffeine. The integration of pretreated SCG is recommended to improve the generation of bioelectricity for sustainable energy applications. Based on these results, it can be concluded that pretreated SCG with aqueous extraction helps to improve the performance of the SMFC system and thus simultaneously supports the reuse of waste.

## Figures and Tables

**Table 1:** Experimental configuration of spent coffee ground (SCG) with and without aqueous extraction in the sediment microbial fuel cell (SMFC).

Cases	SCG (C)	Rinsing (R)	Immerse (I)	Drying (D)
SMFC-Cont	-	-	-	-
SMFC-C	√	-	-	-
SMFC-CD	√	-	-	√
SMFC-CRD	√	√	-	√
SMFC-CRID	√	√	√	√

## Data Availability

Data are contained within this article.
